# Supplementary material for: The Impact of Liver Steatosis on Interleukin and Growth Factors Kinetics during Chronic Hepatitis C Treatment
Source: J Clin Med. 2024 Aug 16;13(16):4849. doi: 10.3390/jcm13164849 (PMC11355301; doi:10.3390/jcm13164849)
Supplement: Supplementary file 1 [file jcm-13-04849-s001.zip › jcm-3136041-supplementary.pdf]

Supplementary Figure 1
Comparison of cytokine and growth factor serum concentrations at four selected time points (before treatment, week 4 and 8 of treatment and at SVR), stratified by the presence of liver steatosis. Shown are medians with IQRs. Repeated measured two-way ANOVA with Tukey's multiple comparisons test was used to calculate the source of variations. *: $p < 0.05$, **: $p < 0.01$, ***: $p < 0.001$.

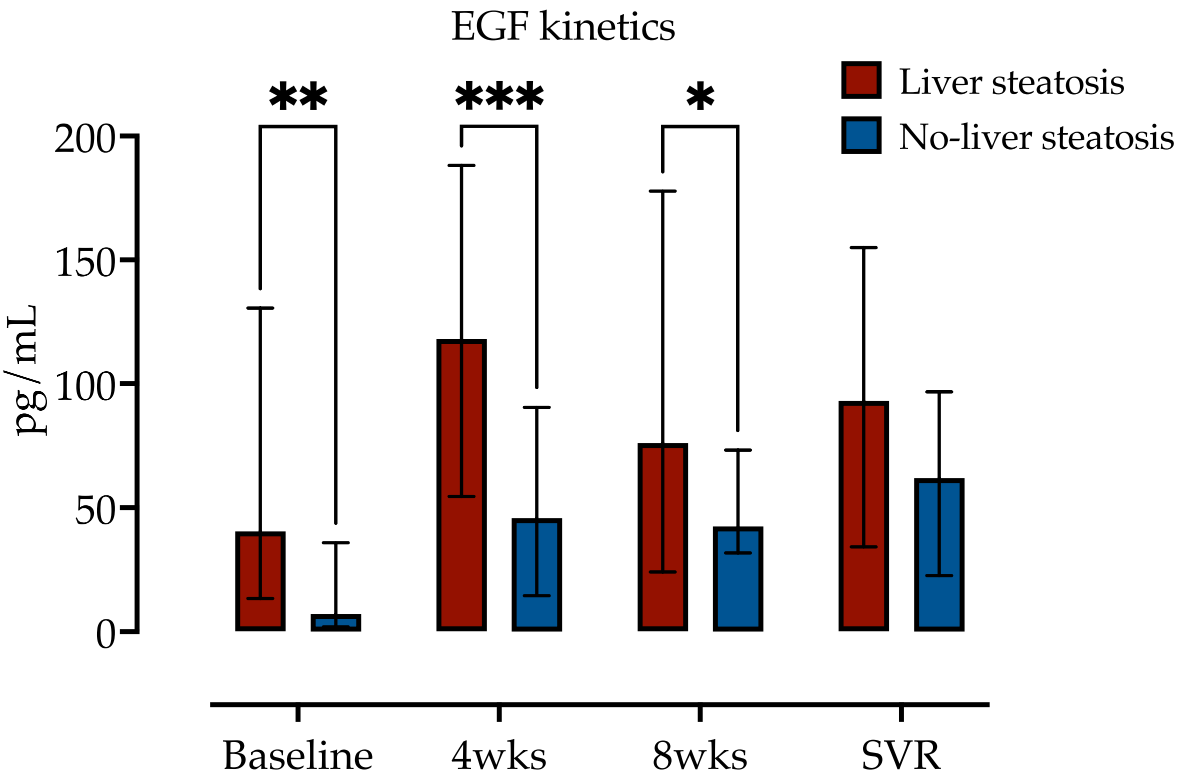

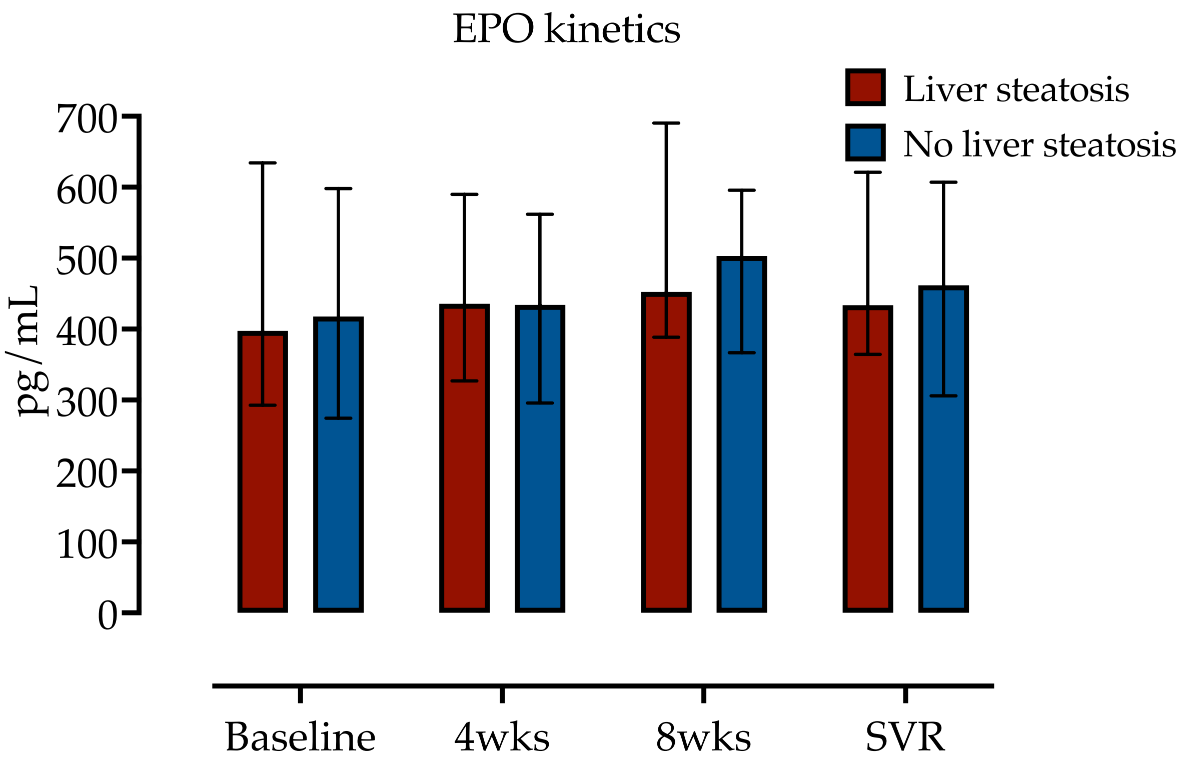

EPO kinetics
Liver steatosis
No liver steatosis
pg/mL
0
100
200
300
400
500
600
700
Baseline
4wks
8wks
SVR

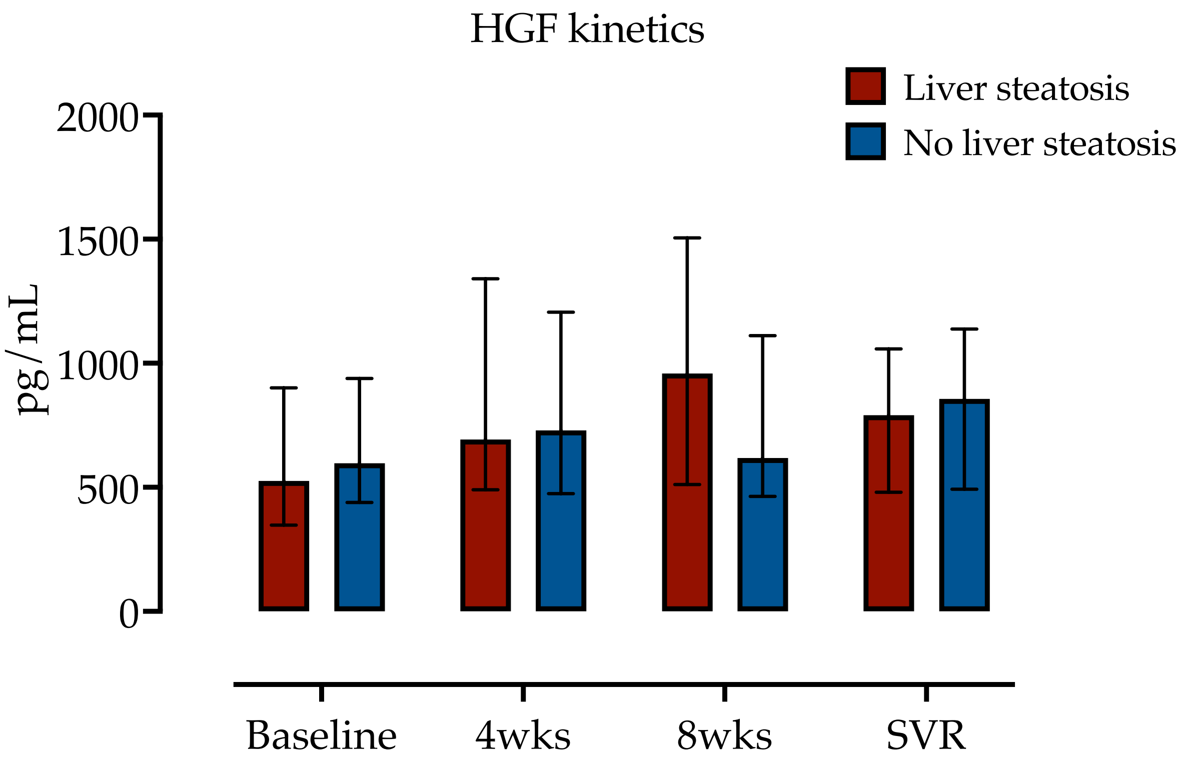
HGF kinetics
Liver steatosis
No liver steatosis
pg/mL
2000
1500
1000
500
0
Baseline
4wks
8wks
SVR

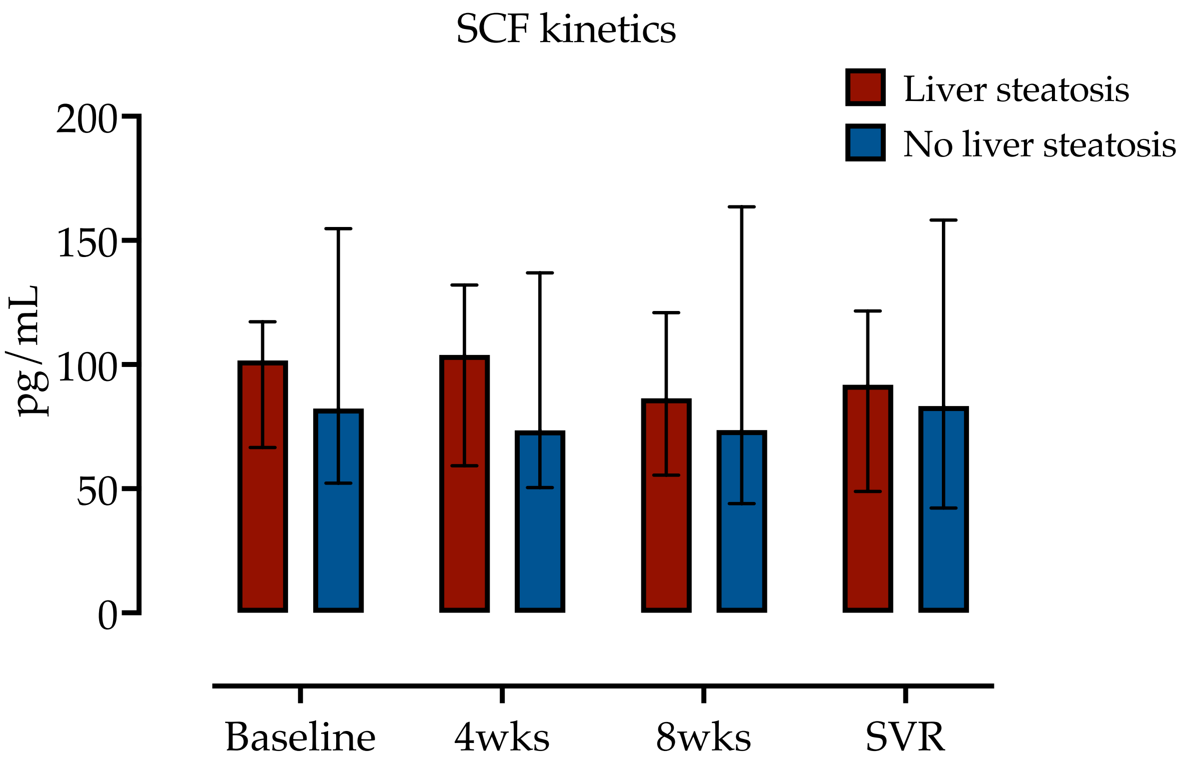

SCF kinetics
Liver steatosis
No liver steatosis
pg/mL
200
150
100
50
0
Baseline
4wks
8wks
SVR

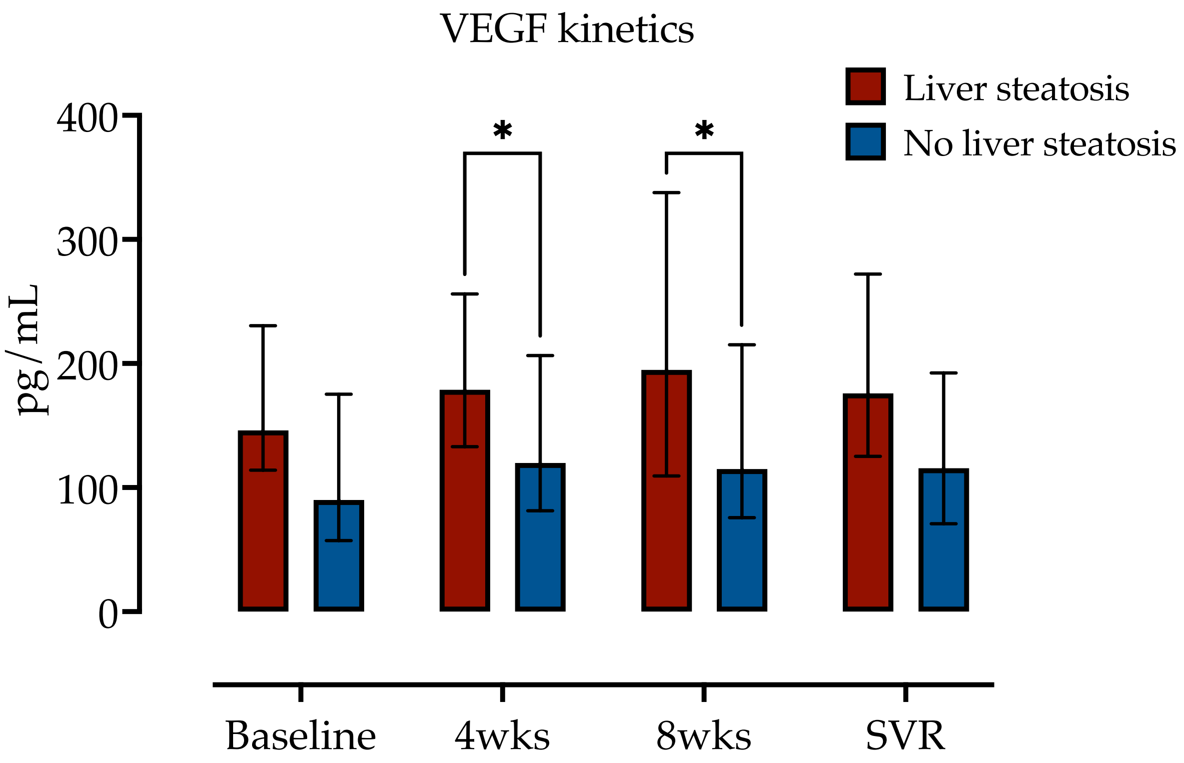

VEGF kinetics
Liver steatosis
No liver steatosis
pg/mL
400
300
200
100
0
*
*
Baseline
4wks
8wks
SVR

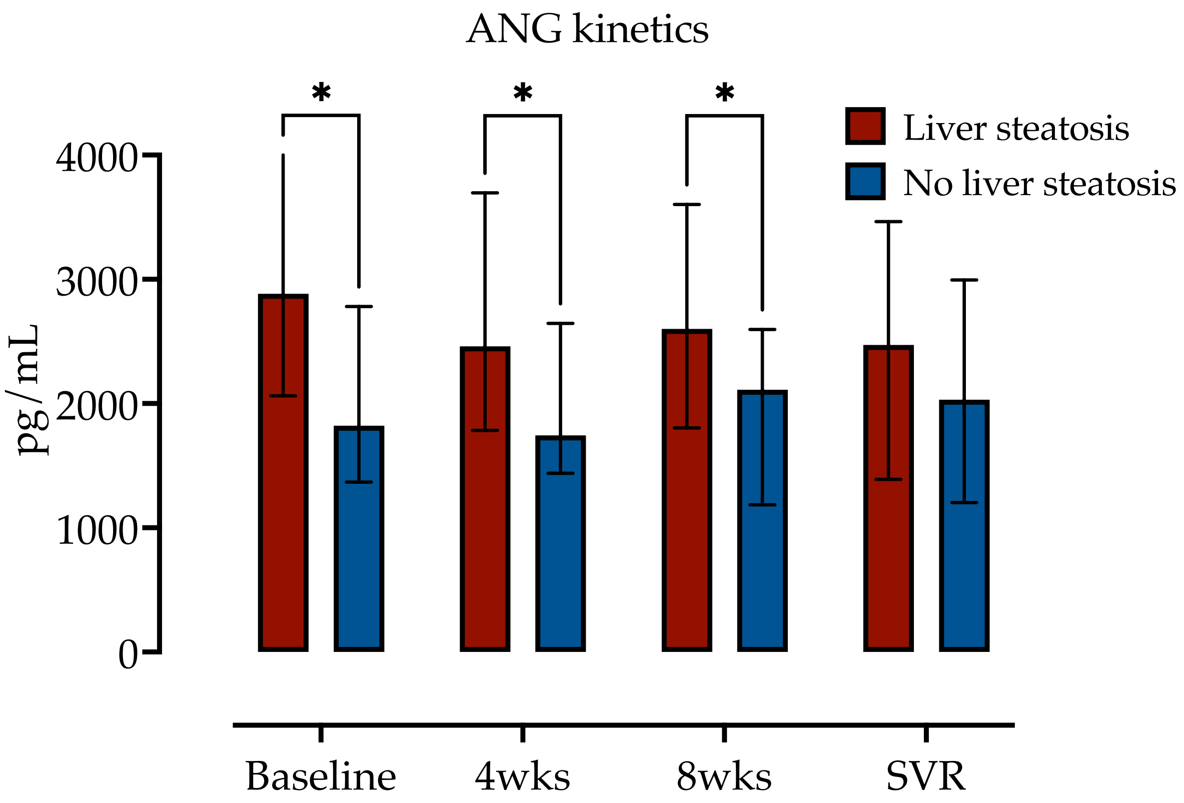
ANG kinetics
Liver steatosis
No liver steatosis
pg/mL
4000
3000
2000
1000
0
*
*
*
Baseline
4wks
8wks
SVR

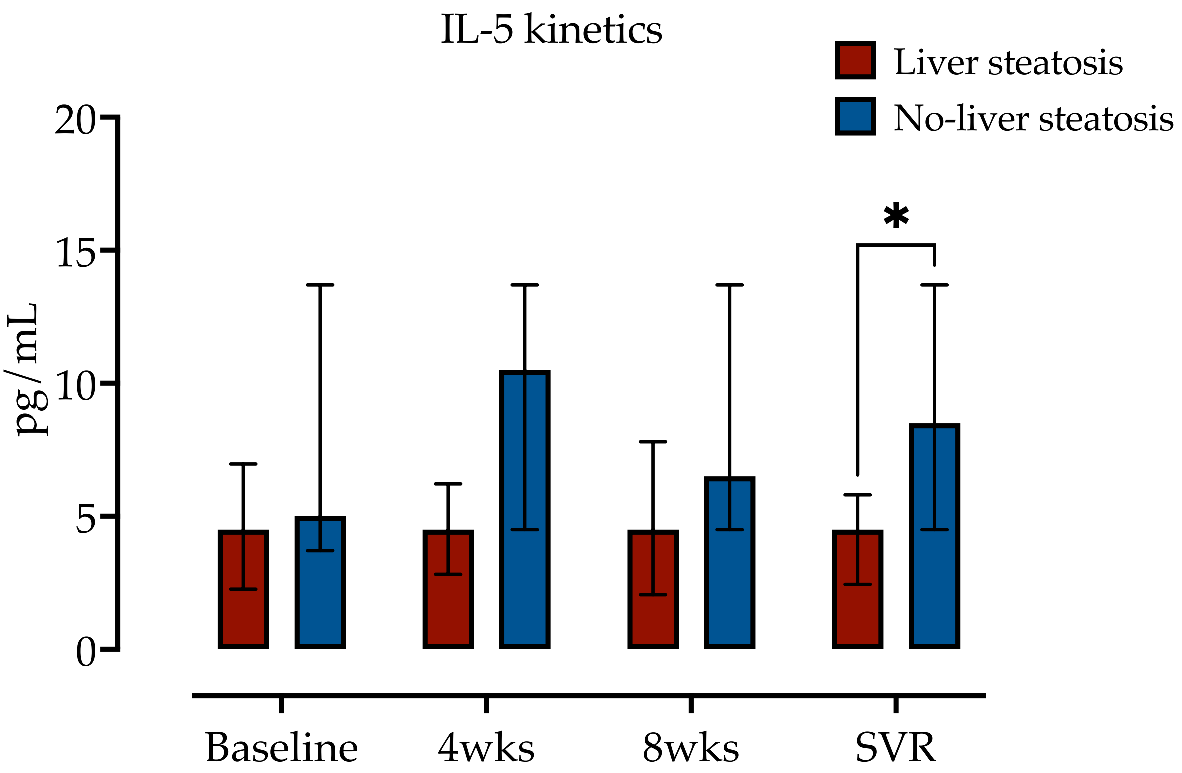

IL-5 kinetics
Liver steatosis
No-liver steatosis
pg/mL
20
15
10
5
0
*
Baseline
4wks
8wks
SVR

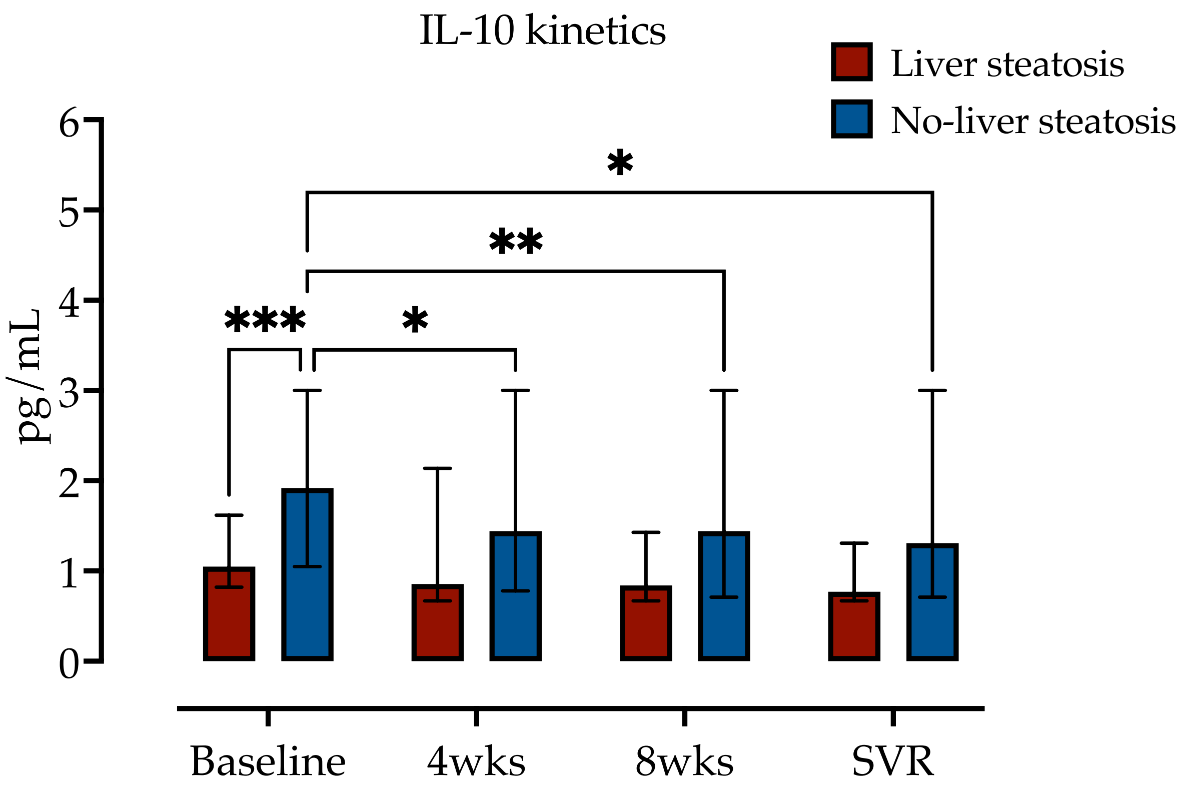
IL-10 kinetics
Liver steatosis
No-liver steatosis
pg/mL
6
5
4
3
2
1
0
***
*
**
*
Baseline
4wks
8wks
SVR

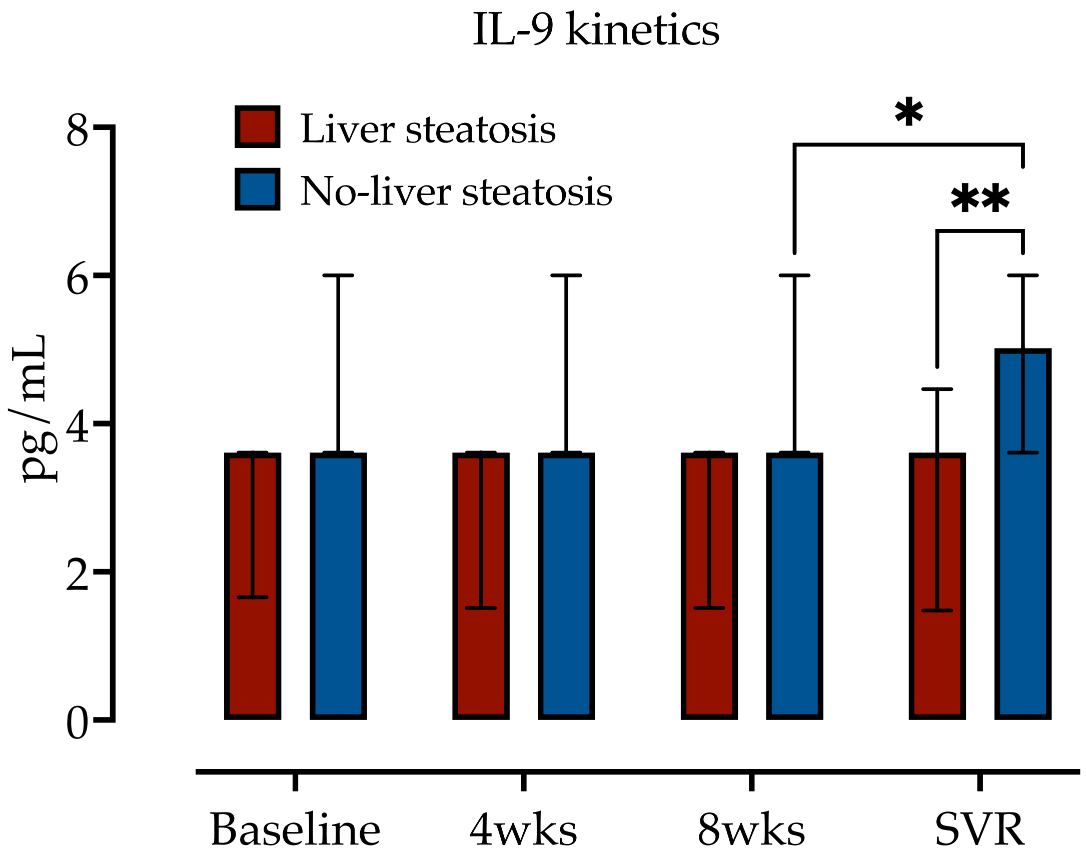
IL-9 kinetics
Liver steatosis
No-liver steatosis
*
**
pg/mL
0
2
4
6
8
Baseline
4wks
8wks
SVR

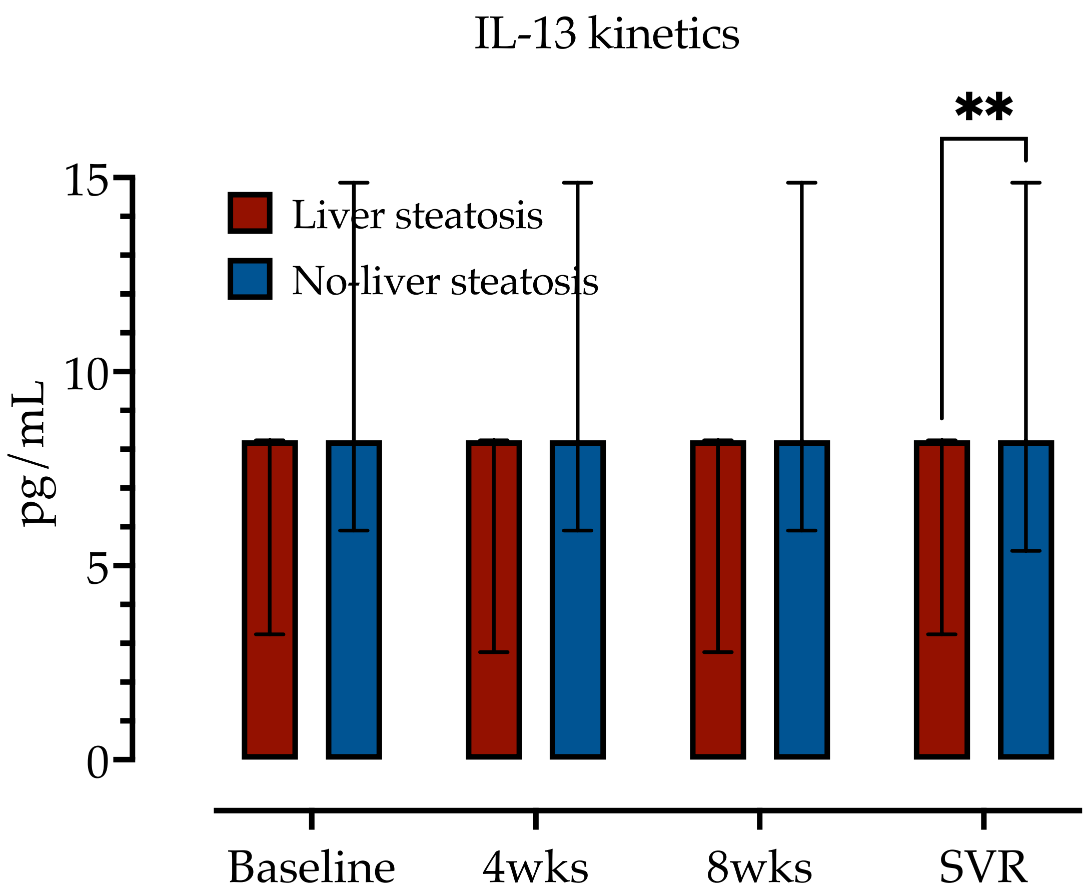
IL-13 kinetics
**
Liver steatosis
No-liver steatosis
pg/mL
15
10
5
0
Baseline
4wks
8wks
SVR

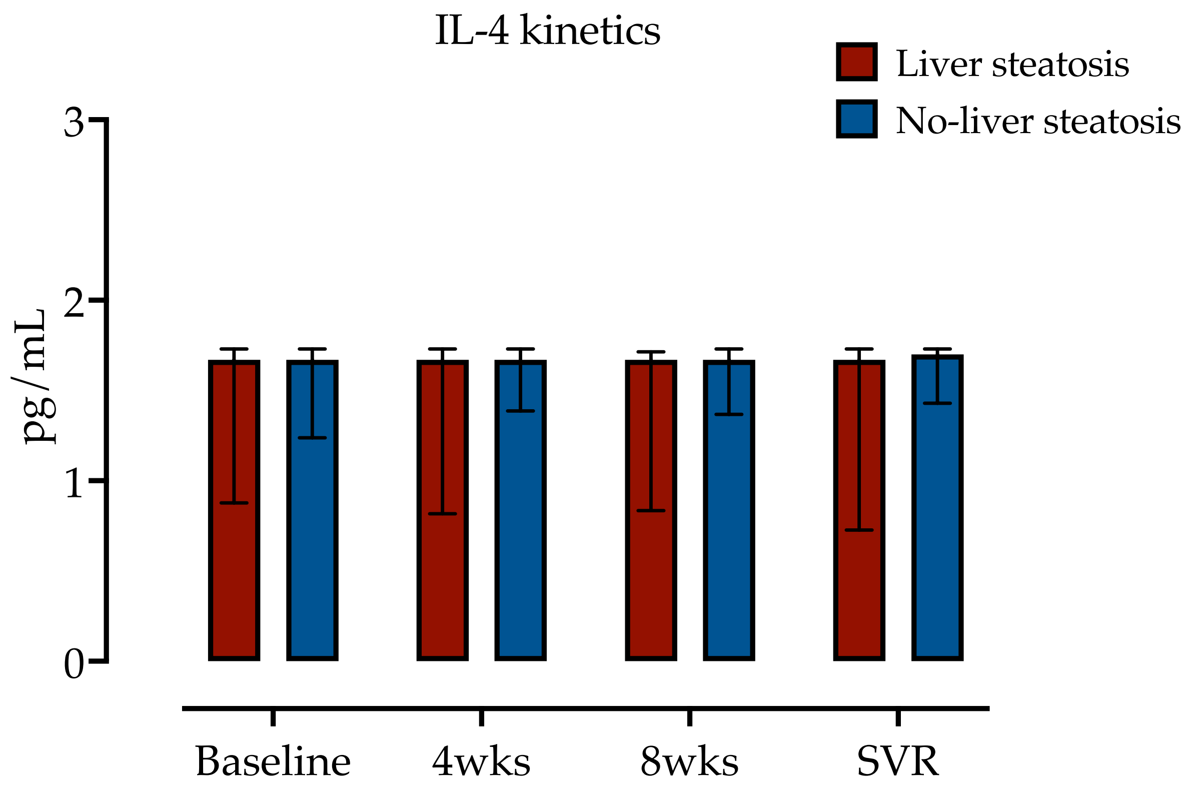
IL-4 kinetics
Liver steatosis
No-liver steatosis
3
2
1
0
pg/mL
Baseline
4wks
8wks
SVR

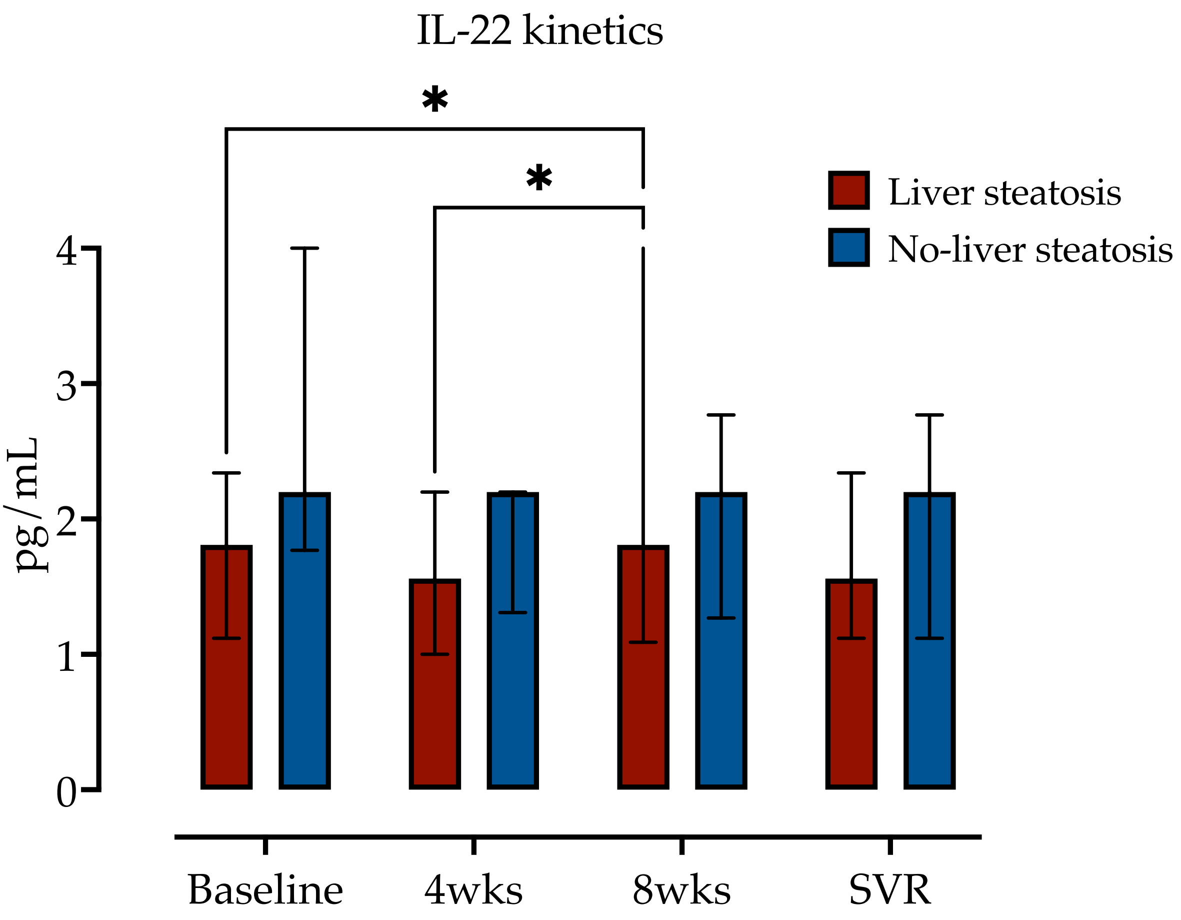
IL-22 kinetics
*
*
Liver steatosis
No-liver steatosis
pg/mL
4
3
2
1
0
Baseline
4wks
8wks
SVR

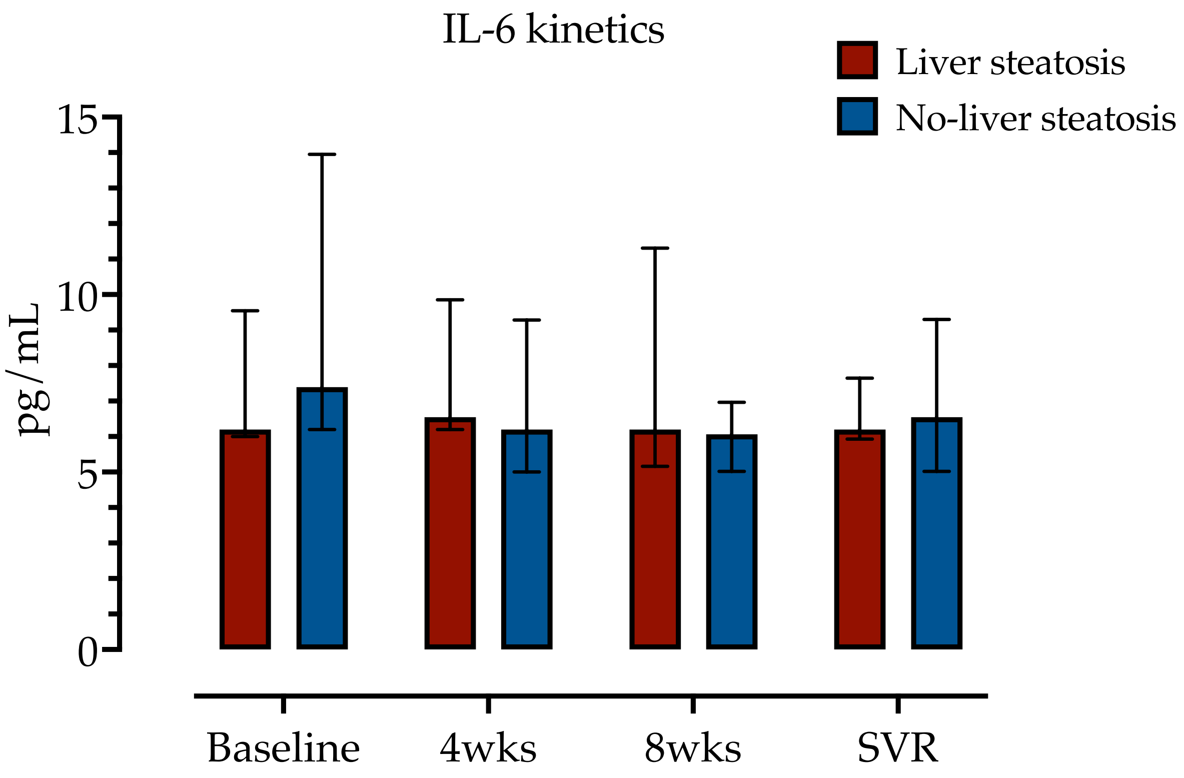
IL-6 kinetics
Liver steatosis
No-liver steatosis
pg/mL
0
5
10
15
Baseline
4wks
8wks
SVR

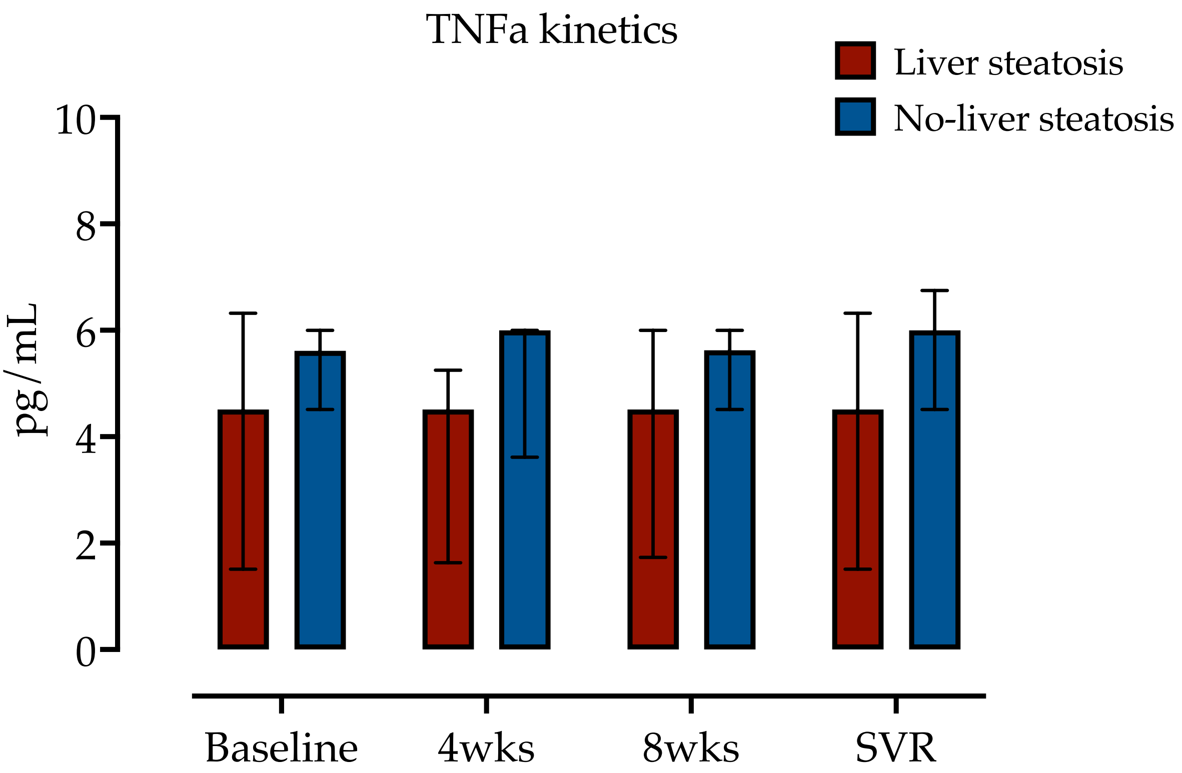
TNFa kinetics
Liver steatosis
No-liver steatosis
pg/mL
10
8
6
4
2
0
Baseline
4wks
8wks
SVR

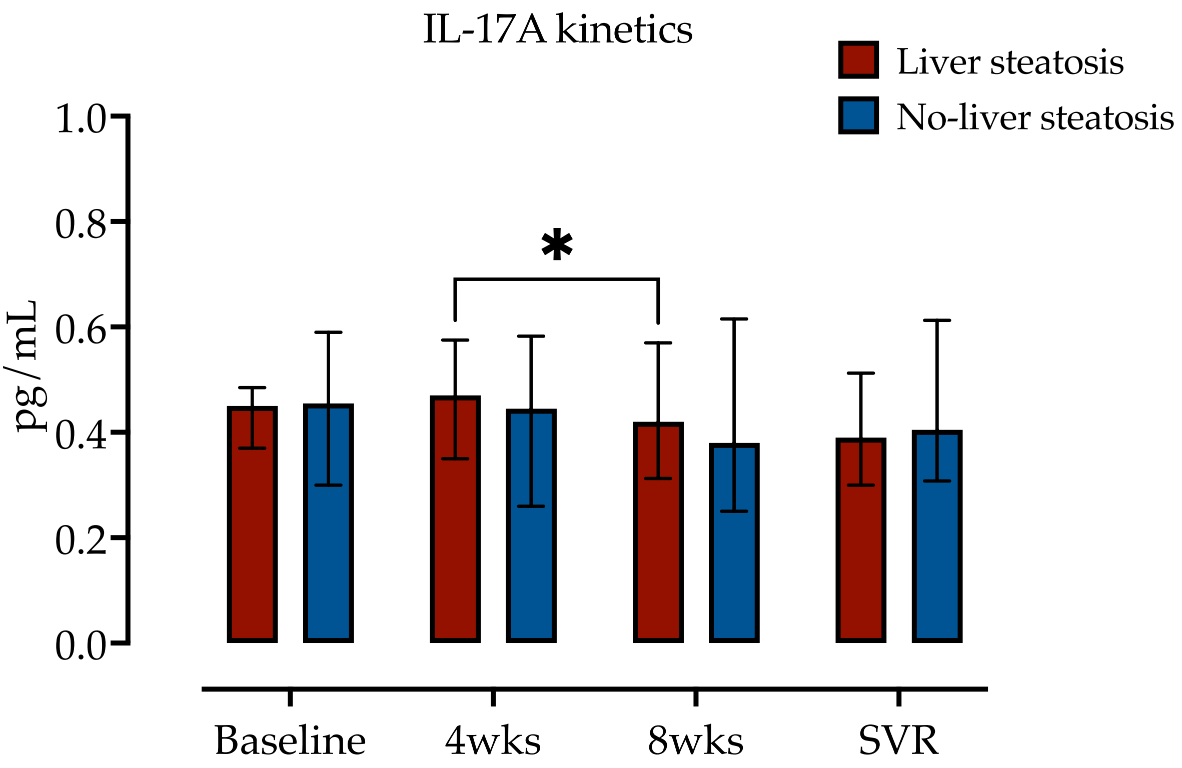

IL-17A kinetics
Liver steatosis
No-liver steatosis
pg/mL
1.0
0.8
0.6
0.4
0.2
0.0
*
Baseline
4wks
8wks
SVR

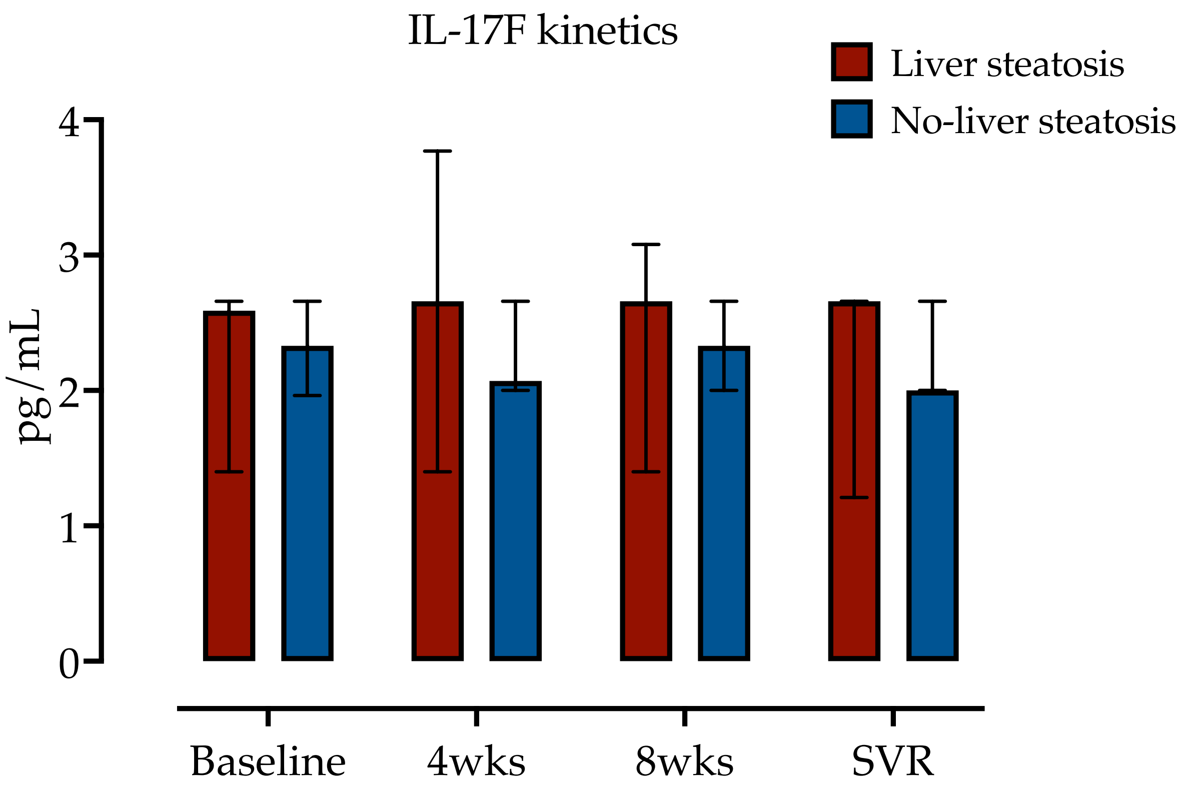
IL-17F kinetics
Liver steatosis
No-liver steatosis
pg/mL
4
3
2
1
0
Baseline
4wks
8wks
SVR

Supplementary Figure 2
Comparison of growth factor and cytokine concentrations at four selected time points (before treatment, week 4 and 8 of treatment and at SVR), stratified by the presence of liver steatosis and liver fibrosis (F0-2 vs F3,4). Shown are medians with IQRs. Repeated measured three-way ANOVA with Tukey's multiple comparisons test was used to calculate the source of variations. Show are p-values,: p < 0.05 is considered significant.

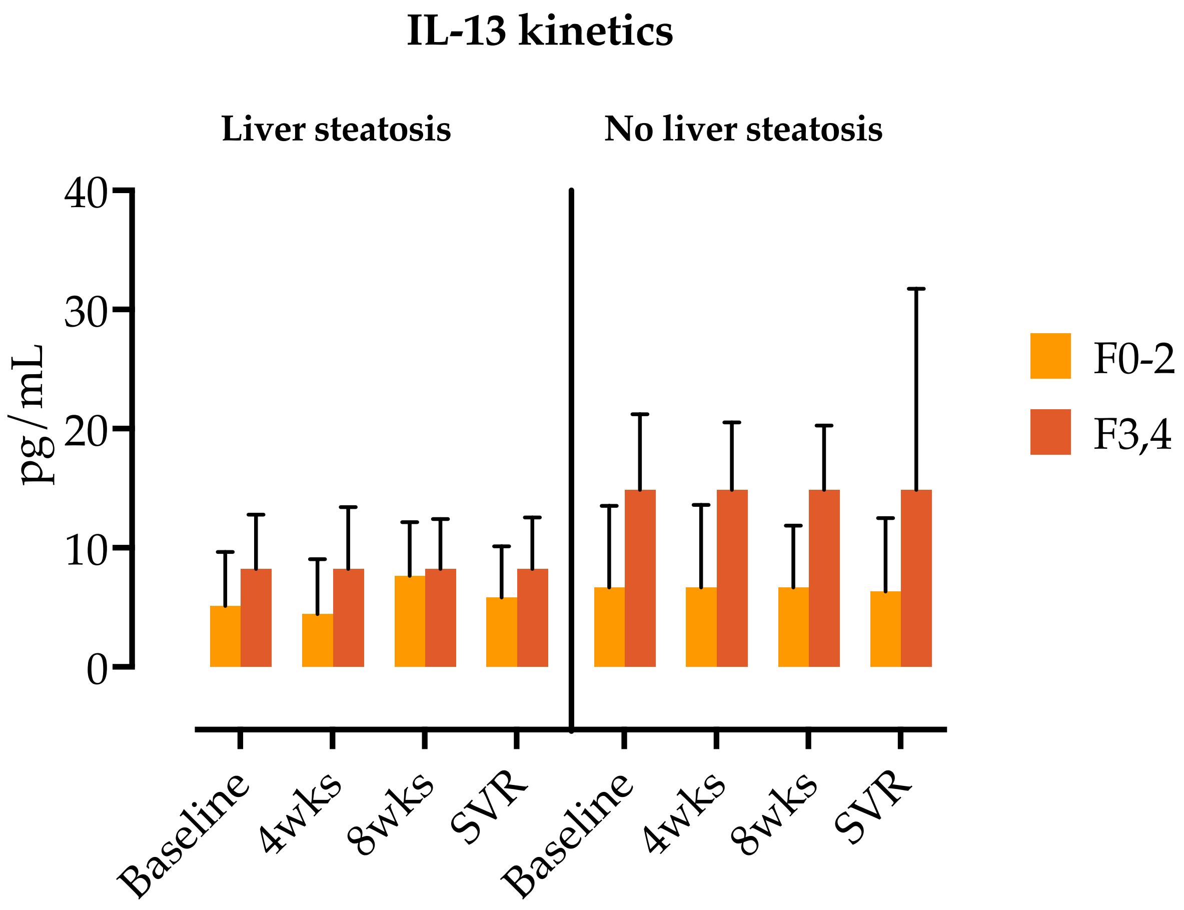

p=0.0364 (Liver steatosis *vs* no steatosis)

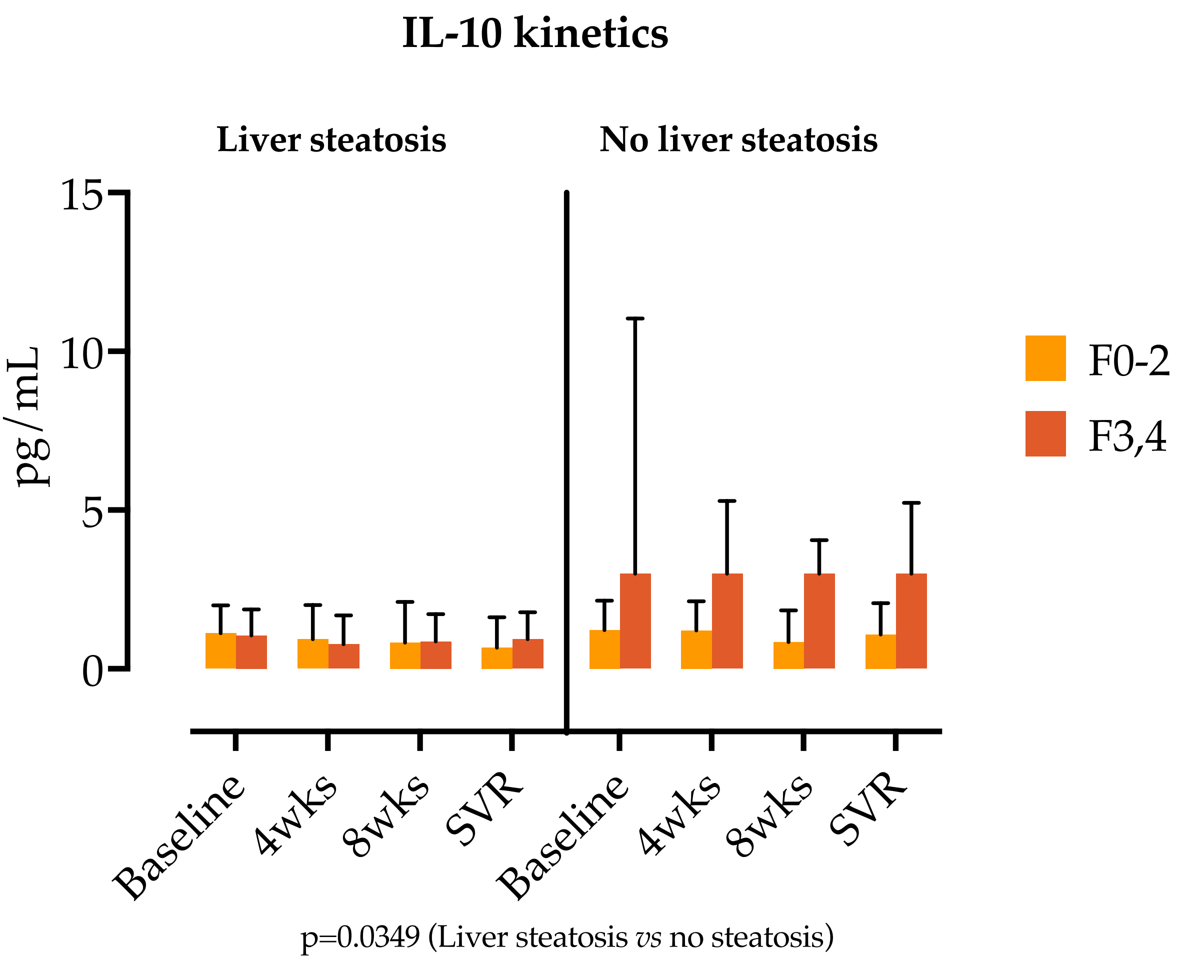
IL-10 kinetics
Liver steatosis
No liver steatosis
pg/mL
15
10
5
0
F0-2
F3,4
Baseline
4wks
8wks
SVR
Baseline
4wks
8wks
SVR
p=0.0349 (Liver steatosis vs no steatosis)

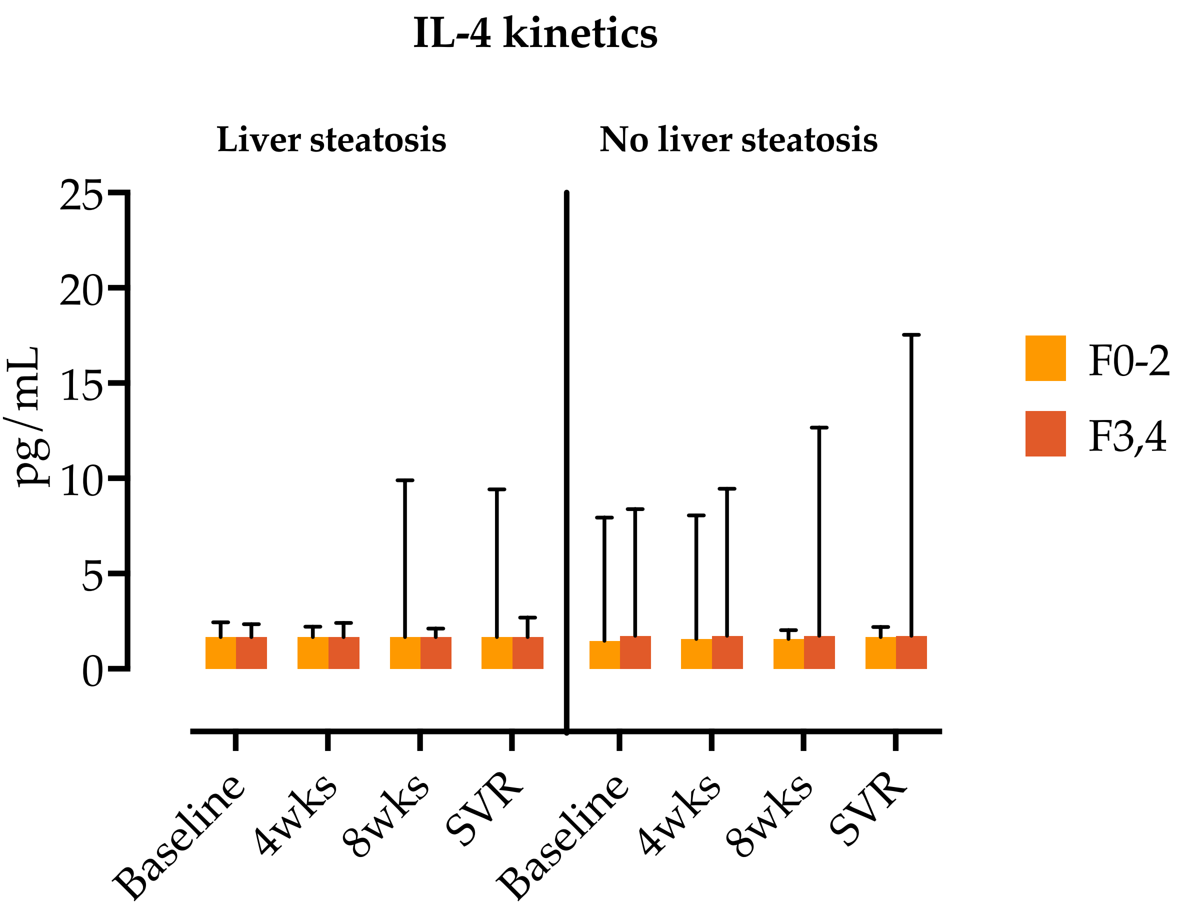

p=0.0107 (Time x (Liver steatosis vs no liver steatosis) x (F0-2 vs F3,4)

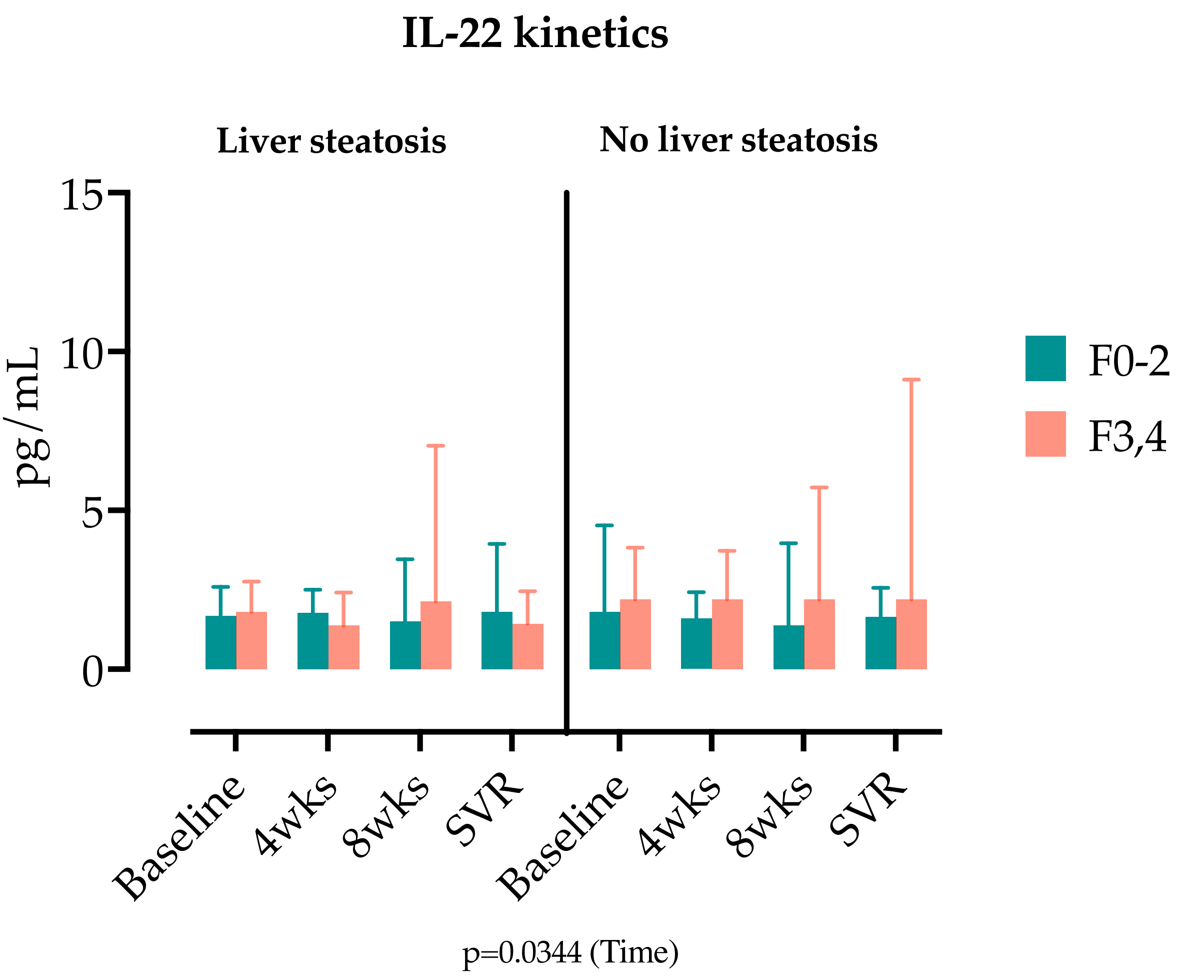
IL-22 kinetics
Liver steatosis
No liver steatosis
15
10
5
0
pg/mL
F0-2
F3,4
Baseline
4wks
8wks
SVR
Baseline
4wks
8wks
SVR
p=0.0344 (Time)

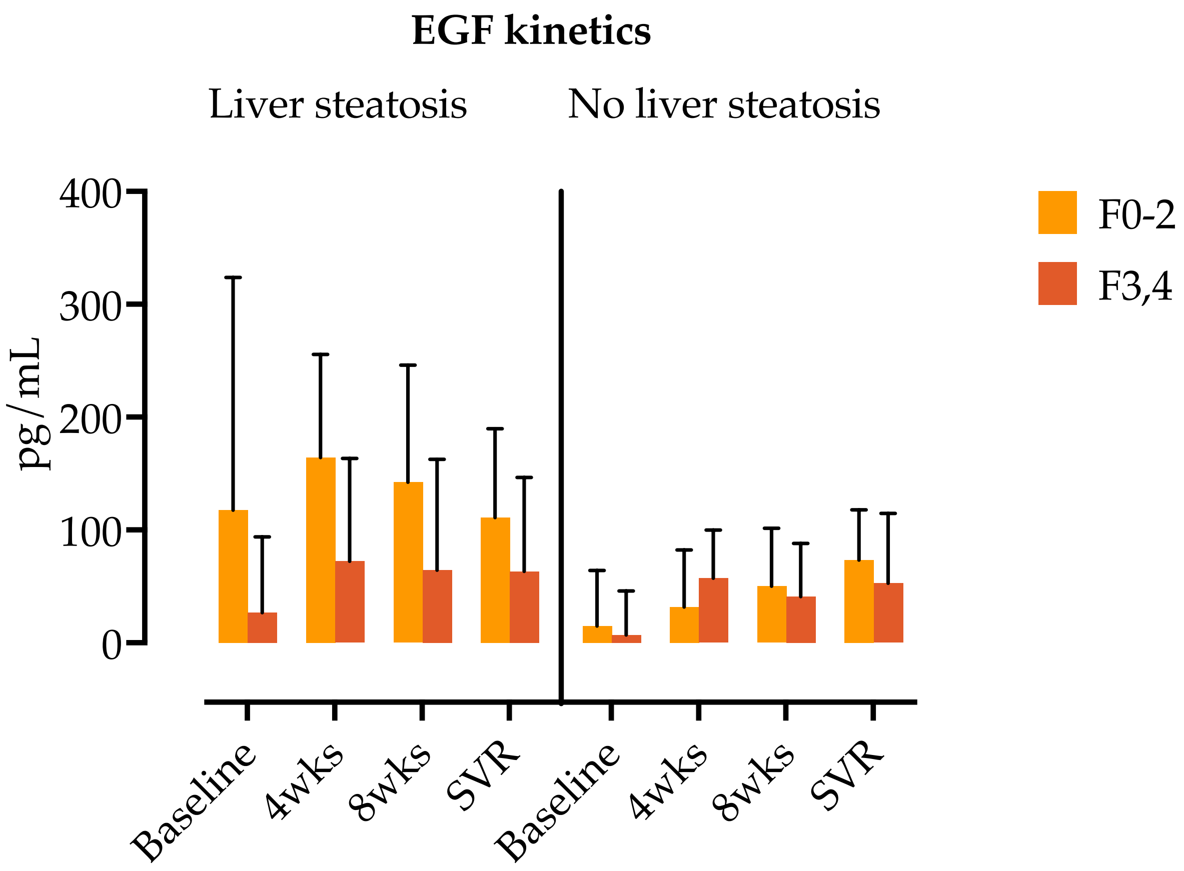

p=0.0003 (Liver steatosis *vs* no steatosis)

VEGF kinetics

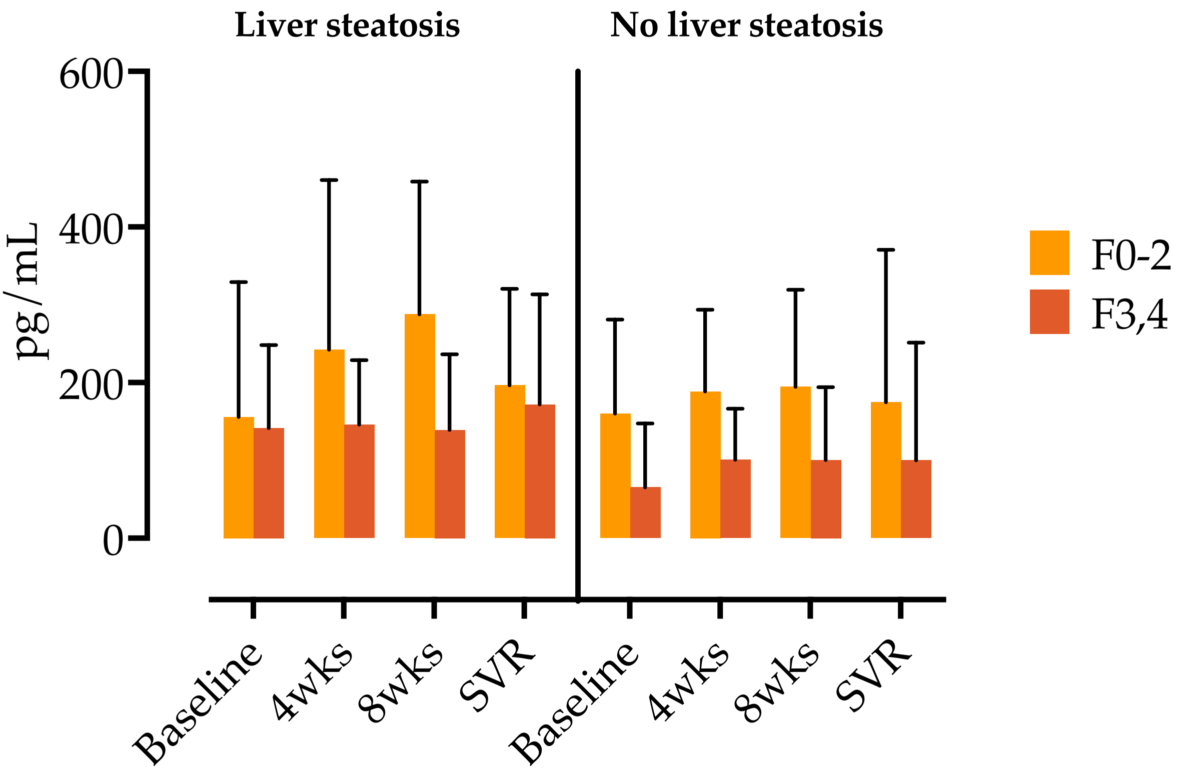

p=0.0193 (liver steatosis vs no steatosis)
p=0.0020 (F0-2 vs F3,4)
p=0.0250 (Time x (Liver steatosis vs no liver steatosis) x (F0-2 vs F3,4)

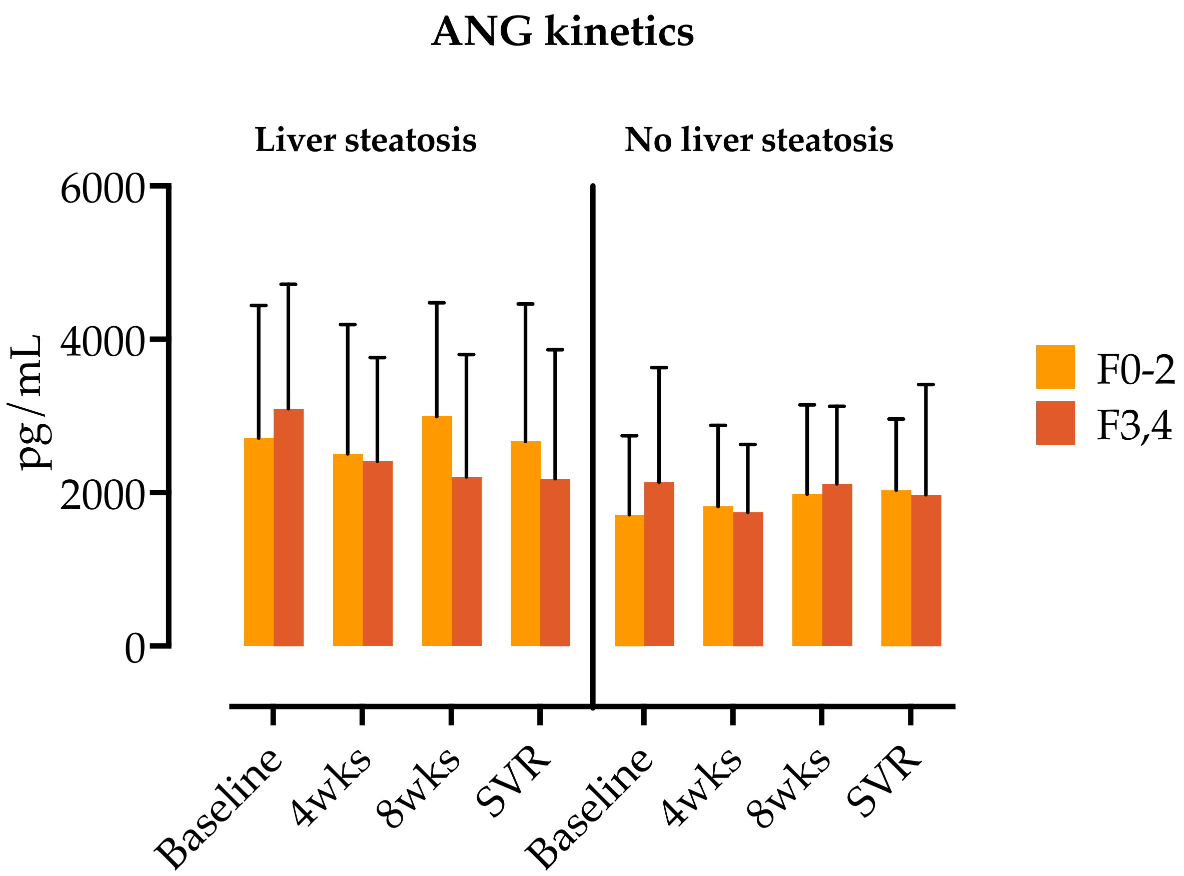

p=0.0182 (Liver steatosis *vs* no steatosis)
